# A Wearable Combined Wrist Pulse Measurement System Using Airbags for Pressurization

**DOI:** 10.3390/s19020386

**Published:** 2019-01-18

**Authors:** Chenling Jin, Chunming Xia, Shiyu Zhang, Liren Wang, Yiqin Wang, Haixia Yan

**Affiliations:** 1 School of Mechanical and Power Engineering, East China University of Science and Technology, Shanghai 200237, China; cljin@mail.ecust.edu.cn (C.J.); syzhang@mail.ecust.edu.cn (S.Z.); wlrleon@hotmail.com (L.W.); 2 School of Basic Medicine, Shanghai University of Traditional Chinese Medicine, Shanghai 201203, China; zyzdx2014@163.com (Y.W.); hjy2012ok@163.com (H.Y.)

**Keywords:** pulse measurement, traditional Chinese medicine, wearable combined wristbands, airbag, position adjustment, pulse-taking pressure, pulse waveform

## Abstract

The pulse measurement instrument is based on traditional Chinese medicine (TCM) and is used to collect the pulse of patients to assist in diagnosis and treatment. In the existing pulse measurement system, desktop devices have large volumes, complex pressure adjusting operations, and unstable pressurization. Wearable devices tend to have no pressurization function or the function to pressurize three channels separately, which are not consistent with the diagnostic method in TCM. This study constructs a wearable pulse measurement system using airbags for pressurization. This system uses guide plates, guide grooves, and positioning screws to adjust the relative position of the wristband and locate Cun, Guan and Chi regions. The pulse signal measured by the sensor is collected and sent to a computer by microcontroller unit. In experiments, this system successfully obtains the best pulse-taking pressure, its pulse waveform under continuous decompression, and the pulse waveform of three regions under light, medium, and heavy pressure. Compared with the existing technology, the system has the advantages of supporting single-region and three-region pulse acquisition, independent pressure adjustment, and position adjustment. It meets the needs of home, medical, and experimental research, and it is convenient and comfortable to wear and easy to carry.

## 1. Introduction

**Figure 1:**
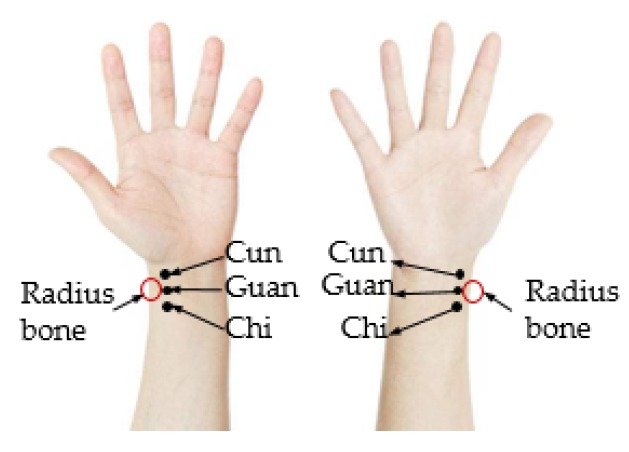
Regions of Cun, Guan and Chi.

In ancient times, during the process of struggling with diseases, Chinese people invented a diagnosis theory called traditional Chinese medicine (TCM) from practices and experiences. TCM contains four diagnostic methods—inspection, listening and smelling examination, inquiry, and pulse taking and palpation [1]. Pulse taking and palpation is the most unique and key diagnostic method. Pulse refers to the beating of the arteries caused by the cardiac output when the heart contracts. According to TCM, pulse condition refers to the speed, intensity, and depth of the pulse. There are three pulse regions of the radial artery in the wrist called Cun, Guan, and Chi, respectively, as is shown in Figure 1. The part slightly below the styloid process of the radius bone is the Guan, one finger anterior the Guan is the Cun, and one finger posterior the Guan is the Chi [2]. The change of pulse condition in each region corresponds to the change of function and state of different organs of the body [3]. For instance, right Cun reflects the functions of the lungs and the large intestine, while left Cun reflects the states of the heart and the small intestine, etc. [4]. TCM physicians use three fingers to locate Cun, Guan and Chi in a patient’s wrist radial artery, adjust the intensity of finger compression, and gain the pulse condition. Based on the results of pulse-taking and the other three diagnostic methods, TCM physicians make diagnoses and treatment plans.

In recent years, as a non-invasive and painless means of detection, pulse-taking has been appreciated and welcomed by people at home and abroad. However, there are some limitations in pulse diagnosis. Firstly, judging pulse by the sensation under the finger is subjective and it is easy to deviate from the actual pulse condition [5]. Secondly, even it takes a great deal of human and material resources to cultivate talents in pulse diagnosis of TCM, the pulse-taking skills are difficult to inherit, which is not conducive to the development and promotion of traditional Chinese medicine. As a result, various research institutes and scholars have investigated the objectivity of pulse-taking [6]. The research usually includes pulse signal acquisition, processing, and classification.

The design of pulse measurement devices is a hot spot of the objectivity research. The key points of the designed device are simulating the modes of pulse diagnosis in TCM and obtaining important information for distinguishing pulse types. There are two modes of pulse measurement. One is to measure the pulse of a single position (usually Guan), and the other is to measure three positions with the theory of Three Positions and Nine Indicators (TPNI). The first is simple and fast while the second is more precise and effective. TPNI plays an important role in pulse diagnosis, and relevant studies have proven the validity of this theory [4,7]. According to TPNI, TCM physicians put three fingers (index, middle, and ring) at Cun, Guan, and Chi, respectively, and apply three different pressures to the three regions, i.e., pressing with light pressure (Fu), pressing with medium pressure (Zhong), and pressing with heavy pressure (Chen). Thus, pulse taking at three positions under three levels of pressure make up nine indicators. Both methods can obtain important information for judging pulse types. There are 28 pulse types in TCM pulse diagnosis. The composition of the pulse condition has four elements—depth, quantity, shape, and strength. Depth refers to the level of pulse depth, quantity refers to the pulse rate, shape refers to the shape of the pulse, and strength refers to the energy of the pulse. Based on these four elements, pulse types can be distinguished. During the process of pulse palpation, physicians find the positions of the region to be measured. Then, they feel the pulse condition and distinguish the pulse type by applying varying static contact pressure (called pulse-taking pressure in TCM) between the fingers and wrist surface. Pulse amplitudes of wrist pulse are different under different pulse-taking pressures. The pulse-taking pressure under which the amplitude of pulse is at maximum is called the best pulse-taking pressure. The best pulse-taking pressure and the trend of pulse amplitude with pulse-taking pressure can be used to judge the depth of pulse, and the pulse waveform can show the quantity, shape, and strength of pulse. Pulse-taking pressure and pulse waveform are significant diagnostic bases. Considering all the factors above, the pulse measurement device should meet the requirement of diagnostic methods in TCM, and the final output of the device should contain pulse-taking pressure and pulse waveform. Therefore, the development of the pulse measurement device should involve sensor selection, pressurizing, position adjusting, overall mechanical structure, and circuit design.

In the last few years, many scholars and research institutes have done research on pulse measurement devices. In terms of sensor usage, most studies use pressure sensors or optical sensors. In general, TCM doctors take the pulse with fingers. The pressure exerted by the fingers and the force of the pulse beat are key factors for judging pulse types. However, pulse measurement devices using optical sensors measure the change of blood flow in the artery, which is different from TCM in measurement principle. The content of the pulse diagrams obtained by the two measurement methods is different. Therefore, the measurement results of optical sensors need to be converted and interpreted before corresponding to the TCM theory. The relationship between the two measurement results requires much research. It is indirect to use optical sensors for measurement. In addition, most current research using optical sensors without pressurization structure renders it impossible to simulate the measurement method of TCM physicians and obtain pulse-taking pressure [2,8,9,10,11]. Consequently, the difference in measurement principle and the lack of pulse information limit the usage of optical sensors in the development of pulse measurement devices. In the aspect of the sensors’ positioning, most existing pulse pressure acquiring devices only contain one channel, resulting in the measurement of one region at a time. When it comes to the devices with multi-channels, because of the complex adjustment mechanism, it takes a long time to locate three regions, and the operation must be guided by a professional. As for the pressurizing mechanism, there are several methods, such as mechanical, pneumatic, and hydraulic. Among these, the mechanical pressurization is the most common one. Mechanical pressurizing devices are usually large in size and heavy in weight, thus it is inconvenient to carry them [12,13,14,15,16]. Finally, most existing wearable devices can only measure heart rate. There are no pressurization parts in these types of devices, thus the static pressure at three regions cannot be adjusted, which leads to the best pulse-taking pressure being unavailable. Thus, the pulse information obtained is insufficient [17,18]. To conclude, the study of the pulse measurement device is of great significance, and there are some problems and defects in the existing equipment that is used to measure pulse that remain to be solved.

This study proposes a wearable combined wrist pulse measurement system that consists of combined wristbands, pressure sensors, pumps and airbags, signal acquisition circuit, signal processing software, a microcontroller unit (MCU), and other parts. The sensors can be easily located to different regions by the mechanism on the wristband. The pressurizing is pneumatic, which reduces the weight and size to make it portable and comfortable to use. It supports two modes; one is measuring the pulse of a single region with a single wristband, and the other is measuring the pulse of three regions simultaneously with combined wristbands. Meanwhile, it can apply various pressures on measuring regions.

## 2. System Architecture and Validation

**Figure 2:**
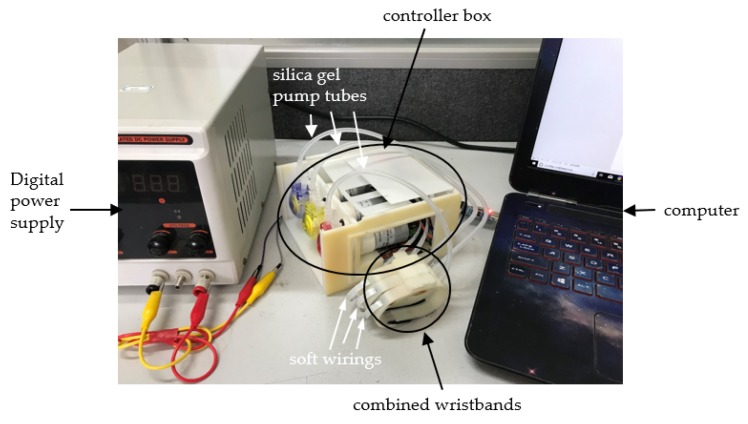
A wearable combined wrist pulse measurement system.

The proposed system consists of combined wristbands and a controller box, as shown in Figure 2. The material of the wristband is rubber 8400 (tensile strength is 2.5 MPa). The thickness of each wristband is 2.2 mm, the length is 200 mm, and the width is 10 mm. The size of the controller box is 130 mm × 130 mm × 90 mm. The total weight of the combined wristbands and the controller box is less than 800 g. The combined wristbands include three separate and identical wristbands. The weight of each wristband is 11 g. Each wristband contains a wrist strap and a stick buckle belt. The position adjustment mechanism is arranged on the upper part of the wrist strap. The airbag is affixed to the inner surface of the wrist strap. The flexible circuit board is arranged in the inner slot of the wristband. The pulse sensor is welded on the circuit board, and it adheres to one side of the airbag. The controller box has an MCU module, a voltage conversion module, and three pumps. The airbags and pumps are connected by silicone tubes. The signal processing circuit board is connected to the MCU by soft wiring.

### 2.1. Signal Acquisition and Processing

**Figure 3:**
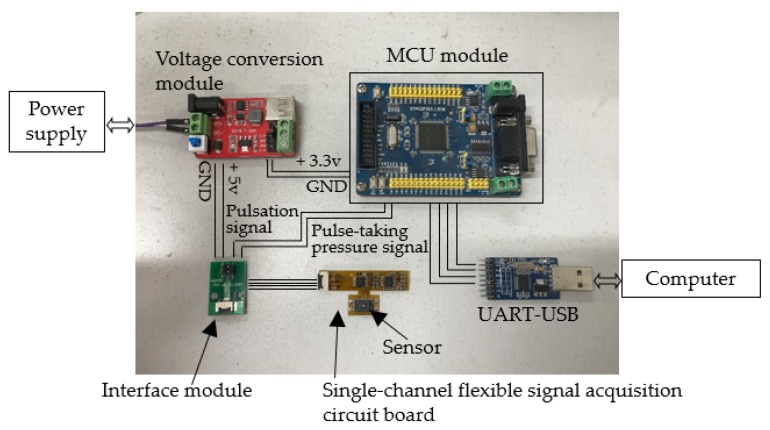
Signal acquisition and processing devices.

The on-wristband circuit and a computer work together to acquire and process pulse signals, as shown in Figure 3. The MCU used in the system is the STM32F103VET6, mainly using Direct Memory Access (DMA) controller, Analog to Digital (A/D) converter, timer, serial port, and other modules. The supply voltage of the MCU is 3.3 V. The depth of the A/D conversion module is 12 bit. The pulse sensor is a force sensor (FSS005WNGB, Honeywell, NJ, USA). Its force sensing range is 0–5 N. The response time is 0.1 ms. The sensitivity is 0.36 mV/g under 5V power supply. Because of the compact size, the sensors can be arranged in array to meet the requirements of the three-region measurement with space for adjusting the position, which is convenient for locating three regions for different people. The accuracy of pressure measurement is calculated by Equation (1):

(1) $$A_{p}=\frac{U_{s}}{2^{R}}\times \frac{1}{S}$$

where $A_{p}$ is the pressure measurement accuracy, $U_{s}$ is the supply voltage of MCU, R is the depth of the A/D conversion module, and S is the sensitivity of the sensor. According to calculation, $A_{p}$ is 0.022 N (2.24 gf). The pressure measurement error of the system is considered to be within ±1.12 gf.

**Figure 4:**
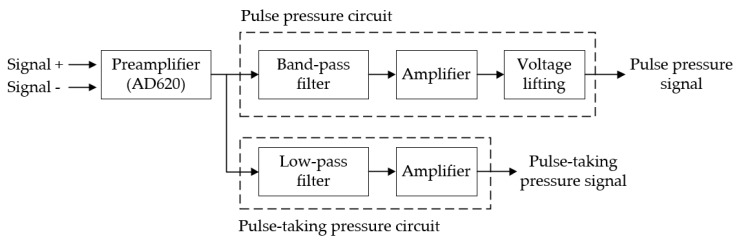
Schematic diagram of signal processing circuit.

Figure 4 shows the block diagram of single-channel signal acquisition and processing devices. First, the sensor signal is amplified, then it is processed by pulse pressure circuit and pulse-taking pressure circuit. The pulse pressure circuit filters, amplifies, and lifts the voltage of the signal and finally outputs the pulse pressure signal. The pulse-taking pressure circuit low pass filters and amplifies the signal. The output is the pulse-taking pressure signal, which is used for calculating the applied pressure that the wrist bears. The theoretical applied pressure can be calculated by Equation (2):

(2) $$F=\frac{1}{A_{s}}\times \frac{U_{\mathrm{pulse}}-U_{0}}{A_{u}}$$

where $U_{\mathrm{pulse}}$ is the pulse-taking pressure signal, $U_{0}$ is the output signal when the input of the pulse sensor is null, $A_{s}$ is the sensitivity, and $A_{u}$ is the gain of pulse-taking pressure circuit.

### 2.2. Principle and Calibration of Pressurization

Each part of the combined wristbands is made of elastic material, which means the wristbands are not only comfortable to wear, but they also make sufficient contact with the surface of the wrist.

**Figure 5:**
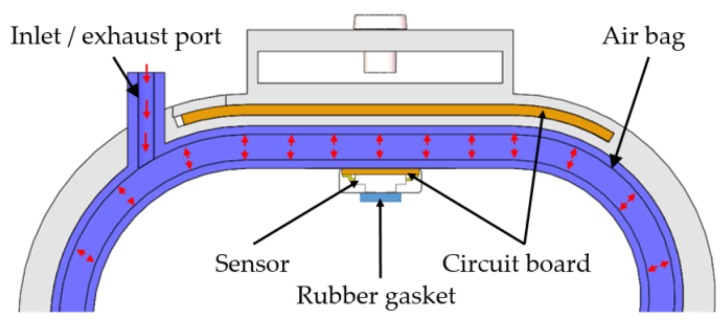
Schematic diagram of pressurization process.

The pumps (KFS-HE, Kamoer, Shanghai, China) fill air into or draw air out of airbags. Thus, the airbags expand or contract, which can apply pressure on the sensors, as shown in Figure 5. Sensors convey the applied pressure to wrist skin through the rubber gasket (diameter is 8 mm) stuck to the sensor. The exchange between air inlet and exhaust and the speed of airflow can be controlled by MCU to adjust the duty circle and rotation direction of the motor. Three air bags are used to pressurize the three sensors, respectively, so the pressure at three regions can be adjusted independently.

**Figure 6:**
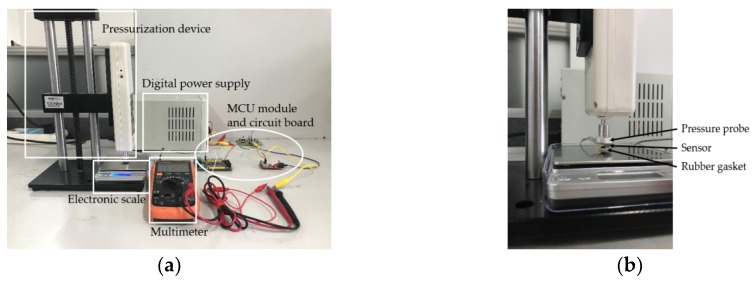
Pressure calibration experiment. (**a**) Experiment devices; (**b**) close view of device.

In the system, the sensor probe contacts the gasket and conveys the pressure to the wrist skin through the gasket. Due to the softness of the gasket, the pressure on the skin is not the same as that measured by the sensors. Besides, in the process of signal processing, the actual gain may vary from the theoretical value. As a result, the relationship between the real pressure and the final output should be figured by conducting a calibration experiment. Figure 6 shows the experiment setup.

**Figure 7:**
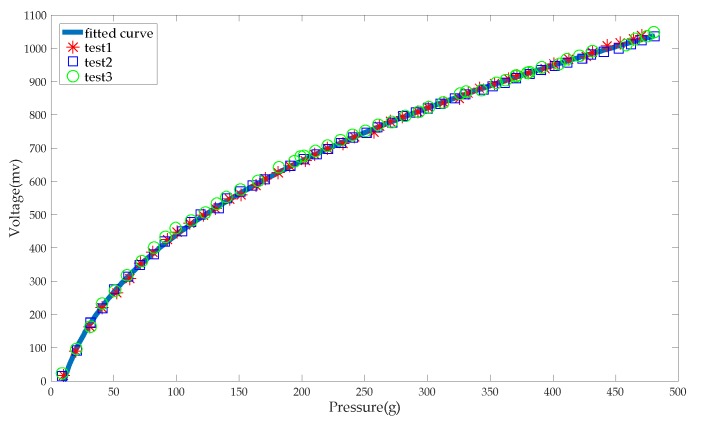
Pressure calibration.

During the calibration experiment, the sensor was pressurized by a pressurization device and conveyed the pressure through the gasket to the electronic scale. The pressure F displayed by the electronic scale was the actual pressure, and the output of the system, i.e., U, was the measured pressure. The range of pressurization was 0 to 480 gf, and the pressure interval was 10 gf. After every 10 gf of pressurization, the stable figures of pressure and voltage were recorded. The experiment was repeated three times. The data points were fitted by power function to find the relationship between the output (electronic scale reading) and the input (pressure on the sensor). The results of calibration and fitting are shown in Figure 7. The correlation between the pressure and system output can be expressed as:

(3) $$U=136.6F^{0.371}-311.5$$

The R-square of the fitting result is 0.9993.

In use, the force is calculated by the output voltage of the system. Therefore, Equation (3) can be expressed as:

(4) $$F=423.9U^{2.075}+19.3$$

where the unit of F is gf, and the unit of U is V. The Root Mean Squared Error (RMSE) is 3.804. Since the value of the force is finally calculated by the calibration formula, the measurement error is mainly caused by the curve fitting. Accordingly, the measurement error is within the range of ± 3.804 gf.

### 2.3. Position Adjusting

**Figure 8:**
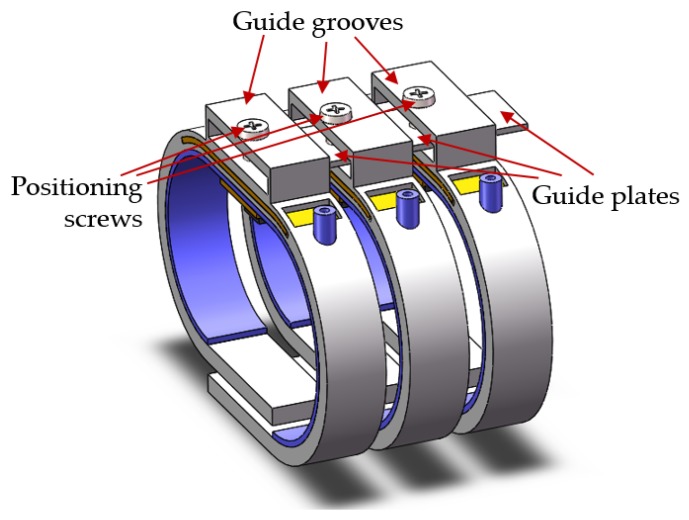
Components and structure of combined wristbands.

TCM physicians adjust positions with three fingers to find Cun, Guan and Chi of patients. The proposed system has three combined wristbands, as shown in Figure 8. The diagnosis position can be located by adjusting the relative distance between the wristbands. The external side of each wristband is provided with a guide groove and a guide plate. Guide plates are inserted into the guiding groove of the adjacent wristband. A positioning screw for fixing the position of the guide plate is arranged at the top of each guide groove.

**Figure 9:**
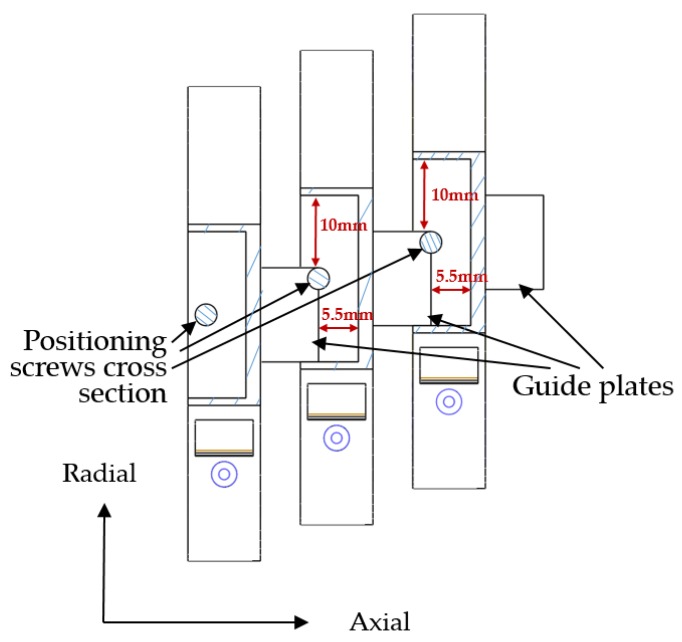
Directions and range of position adjustment in wristbands.

The guide plate moves within the groove to adjust the relative position between the wristband and is then fixed by the positioning screws. The adjusting range of each wristband along the axial is 0–5.5mm and 0–10mm along the radial, as shown in Figure 9.

### 2.4. Validation of the System

TCM physicians take pulse in two ways. One is pressing with one finger, and the other is simultaneous palpation with three fingers. In TCM, basic pulse information like pulse rate, pulse waveform, best pulse-taking pressure, and trend of pulse amplitude with pulse-taking pressure are important factors for judging pulse types. To verify the feasibility of the proposed system, we designed the single-region experiment and the three-region measurement experiment. The former experiment was mainly to acquire basic pulse information under continuous decompression at three regions. The latter experiment was to acquire three-region pulse waveforms under three levels of pressure. The experimental data were collected from 12 healthy students (aged between 23 and 26, five females and seven males) who volunteered to participate in the experiments. The sampling frequency was 1000 Hz, and the range of applied pressure was 0 to 160 gf. To locate the regions of Cun, Guan and Chi, we performed the following steps according to TCM and relevant studies [19]. First, we found the radius bone position and used it as a reference in search of pulses. Within the area beside the radius bone, we searched for the Guan position where the pulse amplitude was relatively large. Cun and Chi were located on the sides of the Guan. They were approximately one finger width away from the Guan position. We searched two areas within two areas, searching for the Cun position and the Chi position where the pulse amplitudes were relatively large.

In the single-region experiment, we measured height and weight and calculated the body mass index (BMI) of each subject. BMI is a commonly used international standard to measure the body’s weight and fitness. Previous studies have shown that BMI information is a major indicator for pulse diagnosis and it must be considered when developing the algorithm of pulse diagnosis [20]. In addition, to verify the reliability of the system, we used a pulse measurement instrument (YX-303, YuWell, Jiangsu, China) to measure the heart rates of subjects after five min of rest. Then, we used the proposed system to measure the pulse condition of each subject in turn. During the experiment, each subject sat on the chair quietly and comfortably. Experiment steps were as follows. First, the subject wore a wristband on his wrist. Then, we moved and rotated the wristband to adjust the position of the sensor. The gasket was rightly aligned to the region (Cun, Guan or Chi) when the software interface displayed the relatively large pulse waveform. Next, we started the device and the pressurization began. To avoid the discomfort of the subject caused by the excessive pressure, the pressurization stopped when the pulse amplitude displayed on the software was small and kept stable for a few seconds. The pulse-taking pressure was mostly within 115–160 gf after pressurization. Finally, the pump rotated slowly in reverse direction for one minute to decrease the static pressure to less than 40 gf. During this decompression process, the system collected and stored the data of dynamic pulse signals and static pressure signals. The experiment was repeated once at Cun, Guan, and Chi in sequence for each subject. To verify the reliability of the measurement results, three subjects’ pulses were measured at the Guan position under continuous decompression for one minute. The experiment was repeated eight times for each subject. Each subject rested for one minute between experiments.

In the three-region experiment, we limited the maximum pressure to 160 g, and the three levels of pressure were 25%, 50%, and 75% of the maximum value, respectively. Before the experiment, we let subject No. 6 wear the combined wristband and adjusted the position of each wristband to make the sensors and gaskets align to three regions. Then, we started the device. Three pumps started to inflate until the pulse-taking pressure reached three levels. At each level, the static pressure was constant for 30 s, and the pulse signals were saved during this process.

## 3. Results

### 3.1. Single-Region Measurement Experiment

**Figure 10:**
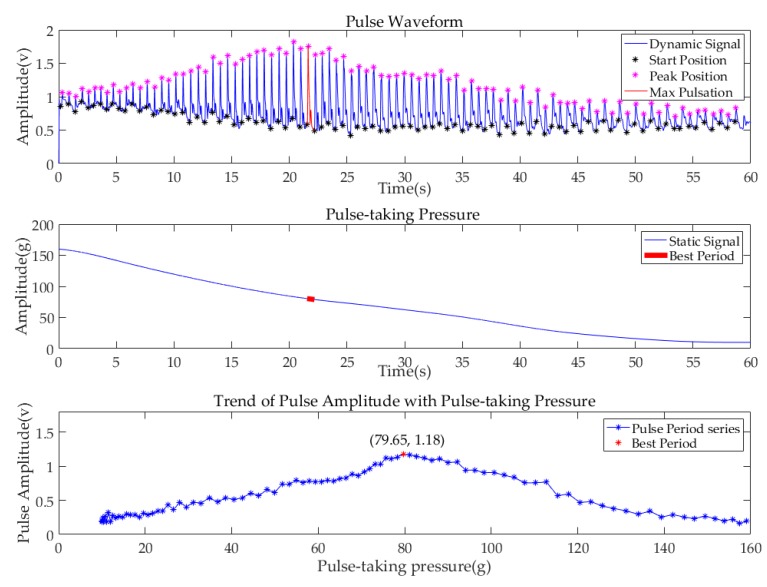
Pulse diagrams of subject (No.6) at the Cun region.

**Figure 11:**
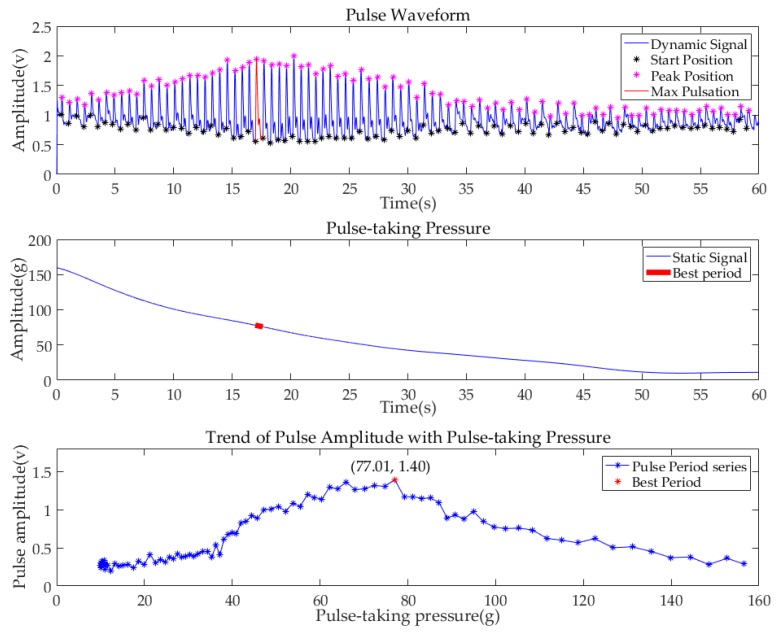
Pulse diagrams of subject (No.6) at the Guan region.

**Figure 12:**
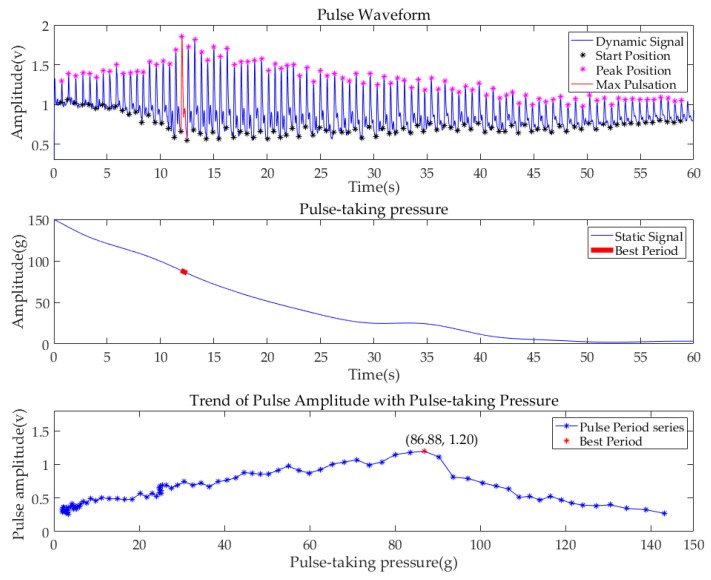
Pulse diagrams of subject (No.6) at the Chi region.

Before further processing, all pulse signals were filtered by the Butterworth filter. The bandpass frequency range was 0.75–20 Hz. To show the performance of the system, the data of the subject (No.6), whose pulse condition was relatively typical, are illustrated in Figure 10. The first diagram shows the pulse waveform of the Cun region in one minute. The start point of each single-period pulse waveform is marked in a black asterisk, while the peak point is marked in a purple asterisk. The second diagram shows the value of pulse-taking pressure of the Cun region in a one min continuous decompression process. By subtracting the amplitude of the starting position from the amplitude of the first peak position in each single pulse period, the maximum pulse amplitude in each single pulse period is obtained. In addition, the pulse-taking pressure of that single period is obtained by averaging the pulse-taking pressure of each single pulse, as shown in the third diagram. The pulse amplitude and the corresponding pulse-taking pressure of all pulse periods in one minute are plotted. The point of the largest amplitude among all periods is marked with a red dot, which is called the best period with the best pulse-taking pressure. Meanwhile, the corresponding pulse period and the static pressure period are marked with red lines in the previous two diagrams. Figure 11 and Figure 12 are the pulse diagrams of Guan and Chi, respectively.

Based on the analysis above, we know that with the decrease of pulse-taking pressure, the pulse amplitudes of three regions first increase and then decrease. The best pulse-taking pressures of subject No.6 are 79.65 gf, 77.01 gf, and 86.88 gf at the Cun, Guan, Chi regions, respectively.

**Figure 13:**
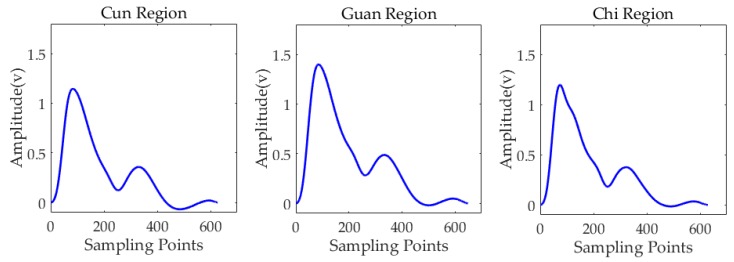
Single period of pulse waveforms under best pulse-taking pressure.

**Figure 14:**
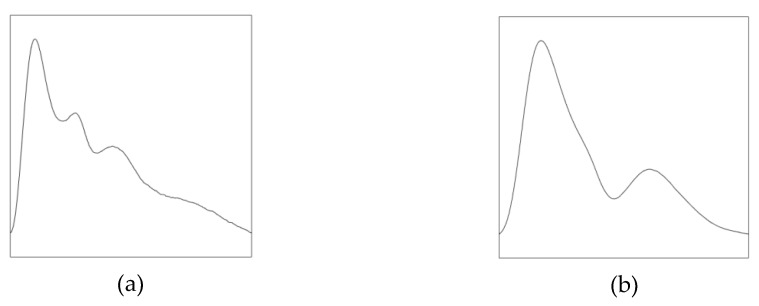
Typical pulse waveforms. (**a**) Normal pulse; (**b**) slippery pulse.

To acquire the pulse waveforms of the best periods, we conducted the experiment for 30 s in three regions under their best pulse-taking pressure. After segmenting multi-period waveforms and calculating the average waveform, the results are shown in Figure 13. Of the 28 types of pulse, there are two types called normal pulse and slippery pulse. Pulse waveforms of the two pulses are shown in Figure 14. Normal pulse has three peaks, while slippery pulse has two peaks. Pulse waveforms of subject No.6 are similar to slippery pulse.

**Figure 15:**
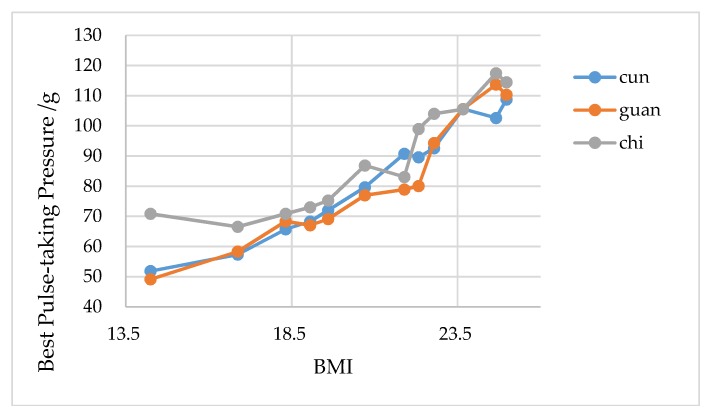
Trend of best pulse-taking pressure in three regions with body mass index (BMI).

All experiment results are shown in Table 1. In terms of heart rate, according to the comparison experiment, the average error of heart rate measurement of the proposed system is less than 2%. Moreover, as clearly shown in Figure 15, the results show that the best pulse-taking pressure of Cun, Guan and Chi are positively correlated with BMI, which means that the best pulse-taking pressure of Cun, Guan, and Chi increases with the increase in BMI. The bigger the BMI, the fatter people tend to be; therefore, these results are consistent with the description within the theory of TCM that "the thin people are more common in floating pulse and the fat people are more common in sunken pulse". Similar conclusions have been drawn in relevant studies [21].

In the validation experiment of measurement, we calculated the average value, standard deviation (SD), and repeatability error (RE) of pulse rate and best pulse-taking pressure of three subjects. The specific analysis results are shown in Table 2. Due to the same measurement method, the results are valid for the Cun position and the Chi position as well. Therefore, the RE of pulse rate is less than 2%, and the RE of best pulse-taking pressure is less than 2.5%. Within these error ranges, measurement errors have little effect on pulse diagnosis.

### 3.2. Three-Region Measurement Experiment

**Figure 16:**
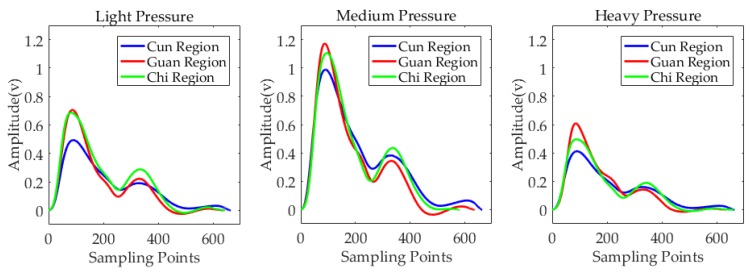
Single period pulse waveforms of three regions under three levels of pressure.

In this experiment, we simulated the diagnostic method of TPNI. Figure 16 shows the single period pulse waveforms of three regions under three levels of pressure. At each level of pressure, the pulse amplitude of the Guan region was higher than the Cun region and the Chi region. Under medium pressure, the pulse amplitudes of three regions were higher than in the other two cases. In TCM pulse diagnosis, the pulse amplitude of floating pulse is larger under light pressure than medium and heavy pressure. For sunken pulse, the pulse amplitude is larger under heavy pressure than the other two levels of pressure. Therefore, according to the preliminary judgment, the pulse types of three positions shown in Figure 16 are neither floating pulse nor sunken pulse.

## 4. Discussions

This study proposed a wearable combined wrist pulse measurement system that integrated a pressurization mechanism. The average error of heart rate measurement is less than 2%. The measurement error is within the range of ±3.804 gf. The RE of system measurement is less than 2.5%. In addition, this system supports single-region measurement and three-region measurement, which are both consistent with the finger palpation technique in TCM. Additionally, positions of wristbands can be adjusted to correspond to the regions of different people. These advantages make the proposed device more suitable and reliable for TCM experiments and research. A series of experiments were done to verify the feasibility of the device. The results show that this system can measure wrist pulse at Cun, Guan and Chi regions and obtain the basic pulse information, including pulse rate, pulse waveform, and the best pulse-taking pressure. The trend of pulse amplitude with pulse-taking pressure is consistent with the description of TCM theories. We also found that there is an increasing trend of the best pulse-taking pressure at three regions with the increase in BMI.

The proposed system and other pulse measurement devices presented in recent studies are summarized in Table 3. Peng Wang proposed a multi-channel pulse signal acquiring system [14]. In Zhou et al.’s study, they proposed a novel three-channel self-pressurized wrist pulse system [12]. Jessica E. T. Kabigting proposed a wearable pulse-taking device [22].

In conclusion, the system is feasible to collect wrist pulse and helpful for TCM diagnosis. Combining the advantages—small volume, light weight, easy to operate, and convenient to carry—it can meet the needs of experimental research and home healthcare. Besides, because of the flexible usage modes of combined wristbands, a variety of combinations are available for the user’s experimental design. In the future, we will improve the function of the system and expand the pressurization range to 200 gf to meet the requirements of different people. In addition, we will study the calibration of the system in the long-term use for users. Furthermore, in order to make the system more accurate and standardized, we will conduct more experiments on the base of TCM. This is of great importance to the objectivity research of pulse diagnosis in TCM.

## Figures and Tables

**Table 1:** Experiment results.

Subject No.	Heart Rate (bpm)		Error (%)	BMI	Best Pulse-Taking Pressure (gf)		
	YX-303	Proposed System			Cun	Guan	Chi
1	96	97	1.04	14.2	51.84	49.11	70.87
2	93	93	0	16.9	57.37	58.37	66.54
3	70	71	1.43	18.3	65.71	68.34	70.82
4	70	71	1.43	19.1	68.30	67.07	73.03
5	87	88	1.15	19.6	72.03	69.12	75.24
6	94	94	0	20.7	79.65	77.01	86.88
7	88	89	1.14	21.9	90.75	78.91	83.07
8	80	79	1.25	22.3	89.57	80.03	98.94
9	73	74	1.37	22.8	92.56	94.31	104.03
10	73	73	0	23.7	105.58	105.55	105.47
11	80	80	0	24.7	102.56	113.70	117.42
12	80	79	1.25	25.0	108.76	110.31	114.49

**Table 2:** Experiment results.

Subject No.	Pulse Rate			Best Pulse-Taking Pressure		
	Average (bpm)	SD (bpm)	RE (%)	Average (gf)	SD (gf)	RE (%)
13	84	0.6	0.73	100.72	1.83	1.81
14	79	1.2	1.53	95.69	2.34	2.45
15	70	1.2	1.72	102.17	2.23	2.18

**Table 3:** Comparisons with other pulse measurement systems.

System	Peng Wang [14]	Zhou [12]	Jessica [22]	Proposed
Wearable wristbands	No	No	Yes	Yes
Combined and detachable	No	No	No	Yes
Position adjustable	Yes	Yes	No	Yes
Pressurization method	Motor and shaft	Motor and shaft	Manually	Pump and air bag
Number of sensors	Sensor array	Three	Three	Three
Sampling frequency	Above 50 Hz	1000 Hz	11 Hz	1000 Hz
Continuous decompression measurement	No	No	No	Yes
Pulse-taking pressure acquiring	Yes	Yes	No	Yes
Year of publication	2012	2015	2017	2019
